# Essential role of connective tissue growth factor (CTGF) in transforming growth factor-β1 (TGF-β1)-induced myofibroblast transdifferentiation from Graves’ orbital fibroblasts

**DOI:** 10.1038/s41598-018-25370-3

**Published:** 2018-05-08

**Authors:** Chieh-Chih Tsai, Shi-Bei Wu, Hui-Chuan Kau, Yau-Huei Wei

**Affiliations:** 1 Department of Ophthalmology, Taipei Veterans General Hospital and National Yang-Ming University, Taipei, Taiwan; 2 Biomedical Commercialization Center, Taipei Medical University, Taipei, Taiwan; 3 Department of Ophthalmology, Koo Foundation Sun Yat-Sen Cancer Center, Taipei, Taiwan; 4 Center for Mitochondrial Medicine and Free Radical Research, Changhua Christian Hospital, Changhua City, Taiwan

## Abstract

Connective tissue growth factor (CTGF) associated with transforming growth factor-β (TGF-β) play a pivotal role in the pathophysiology of many fibrotic disorders. However, it is not clear whether this interaction also takes place in GO. In this study, we investigated the role of CTGF in TGF-β-induced extracellular matrix production and myofibroblast transdifferentiation in Graves’ orbital fibroblasts. By Western blot analysis, we demonstrated that TGF-β1 induced the expression of CTGF, fibronectin, and alpha-smooth muscle actin (α-SMA) in Graves’ orbital fibroblasts. In addition, the protein levels of fibronectin and α-SMA in Graves’ orbital fibroblasts were also increased after treatment with a recombinant human protein CTGF (rhCTGF). Moreover, we transfected the orbital fibroblasts with a small hairpin RNA of CTGF gene (shCTGF) to knockdown the expression levels of CTGF, which showed that knockdown of CTGF significantly diminished TGF-β1-induced expression of CTGF, fibronectin and α-SMA proteins in Graves’ orbital fibroblasts. Furthermore, the addition of rhCTGF to the shCTGF-transfected orbital fibroblasts could restore TGF-β1-induced expression of fibronectin and α-SMA proteins. Our findings demonstrate that CTGF is an essential downstream mediator for TGF-β1-induced extracellular matrix production and myofibroblast transdifferentiation in Graves’ orbital fibroblasts and thus may provide with a potential therapeutic target for treatment of GO.

## Introduction

Graves’ ophthalmopathy (GO) is a cosmetically disfiguring and vision-threatening disease. Although the pathogenesis of GO remains incompletely understood, it is known as an autoimmune inflammatory disease characterized by inflammation, tissue remodeling, expansion, and/or fibrosis^1–3^. In advanced stages of GO, fibrotic changes in orbital tissues may cause restricted ocular motility, proptosis, exposure keratitis, and dysthyroid optic neuropathy, which are often unresponsive to current medical therapies and require surgical intervention^4^. To elucidate the molecular mechanisms that initiate and regulate the process of tissue remodeling and fibrosis in GO is critical for the development of novel treatment strategies.

Multiple studies have established that transforming growth factor-β (TGF-β) play important roles in tissue remodeling and fibrosis via myofibroblast activation in various cell types^5–8^. The protein and mRNA expression levels of TGF-β have been found to be significantly higher in GO orbital tissues and orbital fibroblasts compared with those of normal controls^9–11^. In addition, TGF-β can trigger the differentiation of Thy-1(CD90)-positive orbital fibroblasts into myofibroblasts, identified by an increased expression of alpha-smooth muscle actin (α-SMA)^12^. However, the molecular mechanisms responsible for this elicited response in GO have not been investigated.

Connective tissue growth factor (CTGF), a secreted protein, is thought to be involved in extracellular matrix remodeling during development and pathologic conditions, and can mediate several downstream actions of TGF-β^13,14^. We recently demonstrated that mRNA and protein expression levels of CTGF were significantly increased in GO orbital fibroblasts compared with normal fibroblasts^15^. In addition, the elevated CTGF in GO orbital fibroblasts was associated with the clinical evolution of GO^16^. Noteworthily, it is required to unravel how the upregulation of CTGF contributed to the pathophysiology of GO. In this study, we investigated the role of CTGF in TGF-β-induced extracellular matrix production and myofibroblast transdifferentiation in Graves’ orbital fibroblasts.

## Materials and Methods

### Tissues acquisition and cell culture

The primary cultures of orbital fibroblasts were established from surgical specimens of 4 patients with GO (G1-G4) during decompression surgery (one men and three women; mean age: 37.2 years) and from apparently normal orbital tissues in four age- and sex-matched patients (N1-N4), who received surgery for noninflammatory conditions (one man and three women; mean age: 36.9 years). All specimens were collected in accordance to the Declaration of Helsinki and with informed consent of the patients. All GO patients received methimazole and achieved stable euthyroidism for at least 6 months before surgery, and had been not receiving radiotherapy and not received systemic corticosteroid treatment for at least 1 month before surgery. In addition, all four patients were in inactive stage of GO. The disease activity is evaluated based on the modified clinical activity score^17^. Table 1 showed the clinical characteristics of the four GO patients before surgery. Exclusion criteria include ocular diseases other than GO, pregnancy, as well as individuals suffering from chronic or acute diseases such as diabetes mellitus, hyperlipidemia, diseases of the lung, liver or kidney, cancer, other endocrine dysfunction, and immunological or inflammatory diseases. The surgical orbital tissues were minced aseptically in phosphate-buffered saline (PBS containing 137 mM NaCl, 2.7 mM KCl, 8 mM Na_2_HPO_4_, 1.5 mM KH_2_PO_4_, pH 7.3), and then incubated with a sterile solution containing 130 U/ml collagenase and 1 mg/ml dispase (Sigma-Aldrich Chemical Co., St. Louis, MO, USA) for 24 hours at 37 °C in an incubator filled with an atmosphere of 5% CO_2_^15,18^. The digested orbital tissues were pelleted by centrifugation at 1,000 g, and then resuspended in Dulbecco’s Modified Eagle’s Medium (DMEM, Gibco Life Technologies, Gaithersburg, MD, USA) containing 10% fetal bovine serum (FBS) and a cocktail of antibiotics composed of 100 U/ml penicillin G and 100 μg/ml streptomycin sulfate (Biological Industries, Kibbutz Beit Haemek, Israel). Cultured orbital fibroblasts were used between the 3rd and 6th passages and the cell cultures at the same passage number were used for the same set of experiments. These protocols were approved by the Institutional Review Board of Taipei Veterans General Hospital.

**Table 1:** Clinical characteristics of the four GO patients before surgery in this study.

GO patients	Clinical activity score	Smoking status	Free T4 (0.80–1.90 ng/dL)	T3 (58–159 ng/dL)	TSH (0.40–4.0 μIU/mL)	TSH-receptor Ab (<1.5 U/L)
G1	2	Never	0.97	89	1.014	0.13
G2	1	Never	1.31	96	0.887	1.25
G3	1	Never	1.00	105	0.612	1.12
G4	2	Never	1.25	110	0.693	1.39

### Chemicals and antibodies

The recombinant human TGF-β1 (#P01137) and TGF-β2 (#P61812) were purchased from R&D Systems, Inc. (Minneapolis, MN, USA), and the recombinant human CTGF protein (#PHG0286) was purchased from Thermo Fisher Scientific Inc. (Waltham, MA, USA). The rabbit polyclonal antibodies against the CTGF (#ab6992), Fibronectin (#ab2413) and α-SMA (#ab5694) were acquired from Abcam Inc. (Cambridge, UK). The secondary antibodies against rabbit (#A5795) and mouse (#A9044), and β-actin (#A5441) were purchased from Sigma-Aldrich (St. Louis, MO, USA).

### Western blot analysis

About 1 × 10^7^ cells were trypsinized, pelleted, washed with PBS, and then resuspended in the lysis buffer containing 50 mM Hepes (pH 7.4), 4 mM EDTA, 2 mM EGTA, 1 mM Na_3_VO_2_, 1 mM NaF, 1% Triton X-100, and an aliquot of complete protease inhibitors (Roche Inc., Mannheim, Germany). The suspension was incubated at 4 °C for 20 min and then centrifuged at 10,000 g for another 20 minutes at 4 °C. An aliquot of 50 μg proteins was separated on 10% SDS-PAGE and blotted onto a piece of the PVDF membrane (Amersham-Pharmacia Biotech Inc., Buckinghamshire, UK). After blocking by 5% skim milk in the TBST buffer (50 mM Tris-HCl, 150 mM NaCl, 0.1% Tween 20, pH 7.4) at room temperature for 1 hour, the membrane was incubated for another 1 hour with the primary antibody at room temperature. After washing three times with the TBST buffer, the blot was incubated with a horseradish peroxidase (HRP)-conjugated secondary antibody for 1 hour at room temperature. An enhanced chemiluminescence detection kit (Amersham-Pharmacia Biotech Inc., Buckinghamshire, UK) was used to detect the protein signals with a Fuji X-ray film (Fuji Film Corp., Tokyo, Japan), and the signals were quantified by ImageScanner III with LabScan 6.0 software (GE Healthcare BioSciences Corp., Piscataway, NJ, USA). The expression levels of each protein in the cells from 4 normal subjects and 4 GO patients were normalized by that of corresponding β-actin, respectively (Supplementary data). We define the ratio of CTGF, fibronectin, and α-SMA expression from N1 as 1.0, and thus the relative intensity (folds) from N2-N4 and GO1-GO4 were presented, respectively.

### Analysis of intracellularTGF-β1 content

The intracellular contents of human transforming growth factor-beta 1 (TGF-β1; catalog #DB100B) was quantified in cell culture supernatant by an enzyme-linked immunosorbent assay kit purchased from R&D Systems, Inc. (Minneapolis, MN). Briefly, about 10^5^ orbital fibroblasts were seeded in a 3.5-cm culture dish and incubated for 48 hours at 37 °C in a cell incubator with an atmosphere of 5% CO_2_ followed by treatment of rhCTGF for another 24 hours. According to the manufacturer’s recommendation, cell culture supernatant was centrifuged at 12,000 g at 4 °C, and the aliquots were immediately assayed. The standard for TGF-β1 was used in a range of 0–200 pg/ml, and the results were normalized by the cell number and expressed as pg/10^4^ cells.

### Knockdown of CTGF

The small hairpin RNA (shRNA) plasmids for the CTGF gene (shCTGF) and luciferase control gene (shLuc) were obtained from the RNAi Core Facility at Academia Sinica, Taipei, Taiwan. The shLuc and shCTGF constructs were made by using the pLKO plasmid (http://www.addgene.org/plko) and the target sequences were 5′-CGTGAGTATATTGATCCAGAA-3′ and 5′-GCCCAGACCCAACTATGATTA-3′, respectively. With TurboFect^TM^ *in vitro* transfection reagent (Fermentas Life Science, Vilnius, Lithuania), 2 μg/ml of shCTGF could effectively abolish the CTGF expression in GO orbital fibroblasts at 72 hours according to the protocol recommended by the manufacturer.

### Statistical analysis

Statistical analysis was performed by using the Student’s t test in Microsoft Excel 2016 statistical package and the one-way ANOVA followed by an L.S.D. test in SPSS software. Data are presented as means ± S.D. of the results obtained from three independent experiments. A difference was considered statistically significant between the control and the experimental groups when the p value < 0.05 and p value < 0.01.

## Results

### Up-regulation of the fibrosis-related proteins in GO orbital fibroblasts

**Figure 1:**
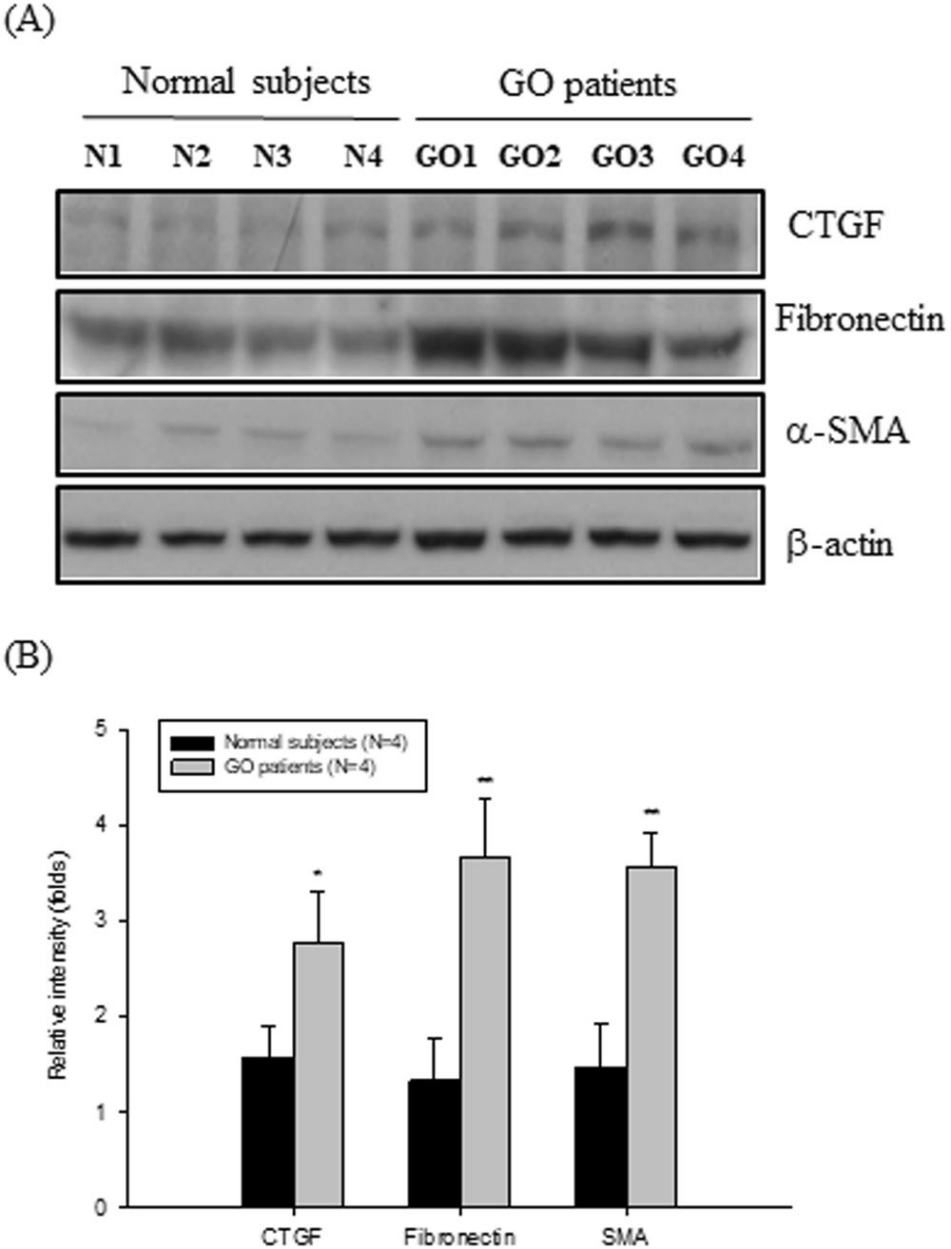
Up-regulation of fibrosis-related proteins in GO orbital fibroblasts as compared to those of normal subjects. (**A**) By Western blots, the fibrosis-related proteins including CTGF, fibronectin and α-SMA were observed in the orbital fibroblasts from four GO patients (GO1-GO4) and four normal subjects (N1-N4). (**B**) By three independent Western blots experiments, we averaged together data from the same normal and patient strain, and then averaged the means of the different normal and patientstrain. The representative histogram was constructed on the basis of the mean values of proteins expression levels in orbital fibroblasts of normal subjects and GO patients, and presented as means ± S.D. (*p < 0.05 vs. normal subjects; **p < 0.01 vs. normal subjects).

By Western blot analysis (detailed in Materials and Methods), we found that the fibrosis-related proteins including CTGF, fibronectin and α-SMA were significantly increased in the primary cultures of GO orbital fibroblasts (GO1-GO4) as compared to those of normal subjects (N1-N4) (p = 0.0232, 0.0083, and 0.0047, respectively) (Fig. 1).

### TGF-β1 induced the fibrosis-related proteins in GO orbital fibroblasts

**Figure 2:**
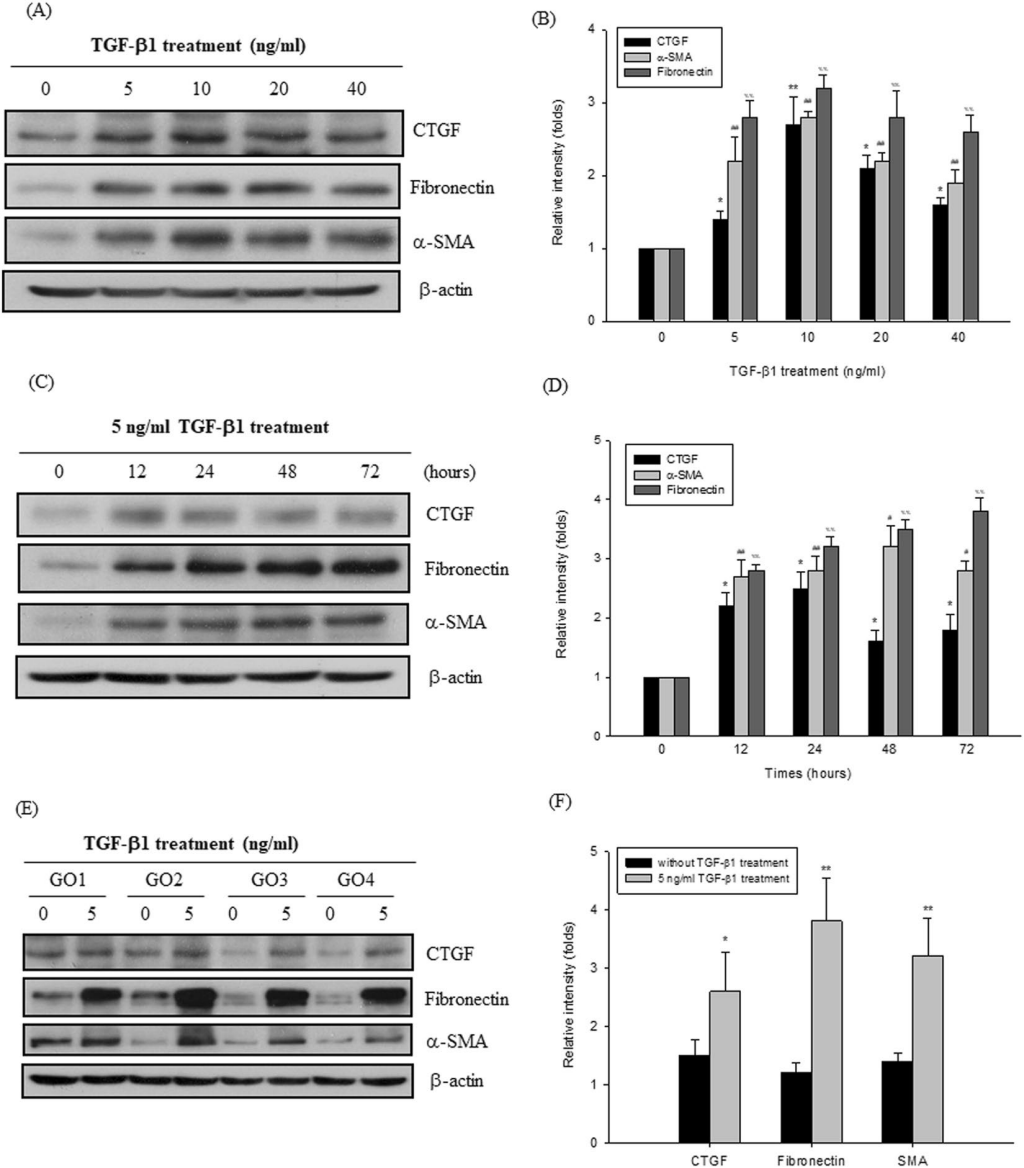
TGF-β1-induced up-regulation of fibrosis-related proteins in the GO orbital fibroblasts. (**A**) After treatment of the orbital fibroblasts from GO1 patient with 5, 10, 20 and 40 ng/ml TFG-β1 for 24 hours, the proteins expression of CTGF, fibronectin, and α-SMA were examined by Western blots, and (**B**) the densitometric scan analysis of three independent Western blots for the fold change of protein expression level was constructed as a bar graphic. Data are presented as means ± S.D. (**C**) After treatment of the orbital fibroblasts from GO1 patient with 5 ng/ml TFG-β1 for 12, 24, 48 and 72 hours, the proteins expression of CTGF, fibronectin and α-SMA were examined by Western blots. (**D**) The representative bar graphic was constructed from three independent experiments, and data are presented as means ± S.D. (**E**) After treatment of the GO orbital fibroblasts from four GO patients (GO1-GO4) with 5 ng/ml TFG-β1 for 24 hours, the proteins expression of CTGF, fibronectin and α-SMA were examined by Western blots, respectively. (**F**) The ratio of CTGF, fibronectin, and α-SMA expression to each β-actin control from GO1 without TGF-β1 treatment were defined as 1.0, and thus the other relative intensity (folds) were presented, respectively. By three independent Western blots experiments, we averaged together data from the same patient strain, and then average the means of the different patient (GO1-GO4, N = 4) strains. The representative histogram was constructed on the basis of the mean values of proteins expression levels in orbital fibroblasts of 4 GO patients, and presented as means ± S.D. (*p < 0.05 vs. control without TGF-β1 treatment; **^,##,%%^p < 0.01 vs. control without TGF-β1 treatment).

**Figure 3:**
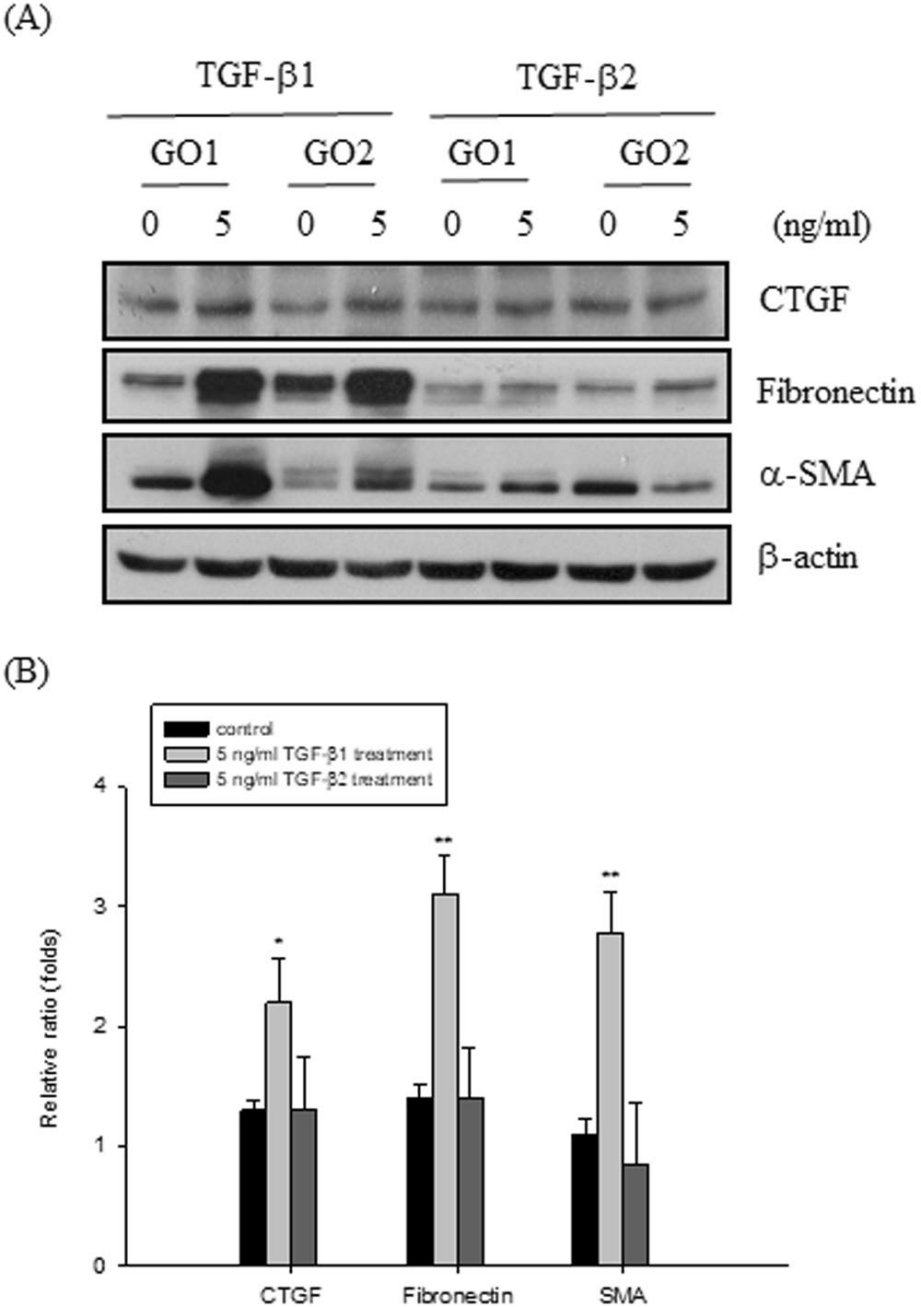
TGF-β1, but not TGF-β2 induced up-regulation of fibrosis-related proteins in the GO orbital fibroblasts. (**A**) After treatment of the GO orbital fibroblasts with 5 ng/ml TFG-β1 and TFG-β2, respectively for 24 hours, the proteins expression of CTGF, fibronectin and α-SMA were examined by Western blots. (**B**) The ratio of CTGF, fibronectin, and α-SMA expression to each β-actin control from GO1 without TGF-β1 treatment were defined as 1.0, and thus the other relative intensity (folds) were presented, respectively. By three independent Western blots experiments, we averaged together data from the same patients strain (GO1-GO4, N = 4), and then averaged the means of the different patients strain. The representative histogram was constructed on the basis of the mean values of proteins expression levels in orbital fibroblasts of 4 GO patients, and presented as means ± S.D. (*p < 0.05 vs. control without TGF-β1 treatment; **p < 0.01 vs. control without TGF-β1 treatment).

Since TGF-β is important for the induction of tissue remodeling and fibrosis, we then observed whether TGF-β1 or TGF-β2 could induce the fibrotic process in the primary cultures of orbital fibroblasts from GO patients. After treatment of the orbital fibroblasts from GO1 patient with 5, 10, 20, and 40 ng/ml TFG-β1, respectively, for 24 hours, the protein levels of CTGF, fibronectin and α-SMA were substantially increased (Fig. 2A,B). In addition, we also observed that the protein expression levels of CTGF, fibronectin and α-SMA were induced in the orbital fibroblasts from GO1 patient by treatment of 5 ng/ml TFG-β1 for 12, 24, 48, and 72 hours, respectively (Fig. 2C,D). The induction of fibrosis-related proteins by 5 ng/ml TGF-β1 treatment for 24 hours was examined in four primary cultures of orbital fibroblasts from GO patients (GO1-GO4). By densitometric analysis, the expression levels of CTGF, fibronectin, and α-SMA were normalized to the corresponding β-actin expression level, respectively. We define the ratio of CTGF, fibronectin, and α-SMA expression to each β-actin control from GO1 without TGF-β1 treatment as 1.0, and thus the other relative intensity (folds) were presented, respectively. By three independent Western blots experiments, we averaged together data from the same patient strain, and then average the means of the different patient (GO1-GO4, N = 4) strains. The average induction folds for CTGF, fibronectin and α-SMA were 1.72 ± 0.22 (p = 0.0251), 3.10 ± 0.37 (p = 0.0067), and 2.42 ± 0.27 (p = 0071), respectively (Fig. 2E,F). However, TGF-β2 could not exert similar effects on the induction of fibrotic proteins in the GO orbital fibroblasts (p = 0.5143, 0.5881, and 0.4371, respectively) (Fig. 3).

### CTGF is critical for the induction of fibronectin and α-SMA proteins expression in GO orbital fibroblasts

**Figure 4:**
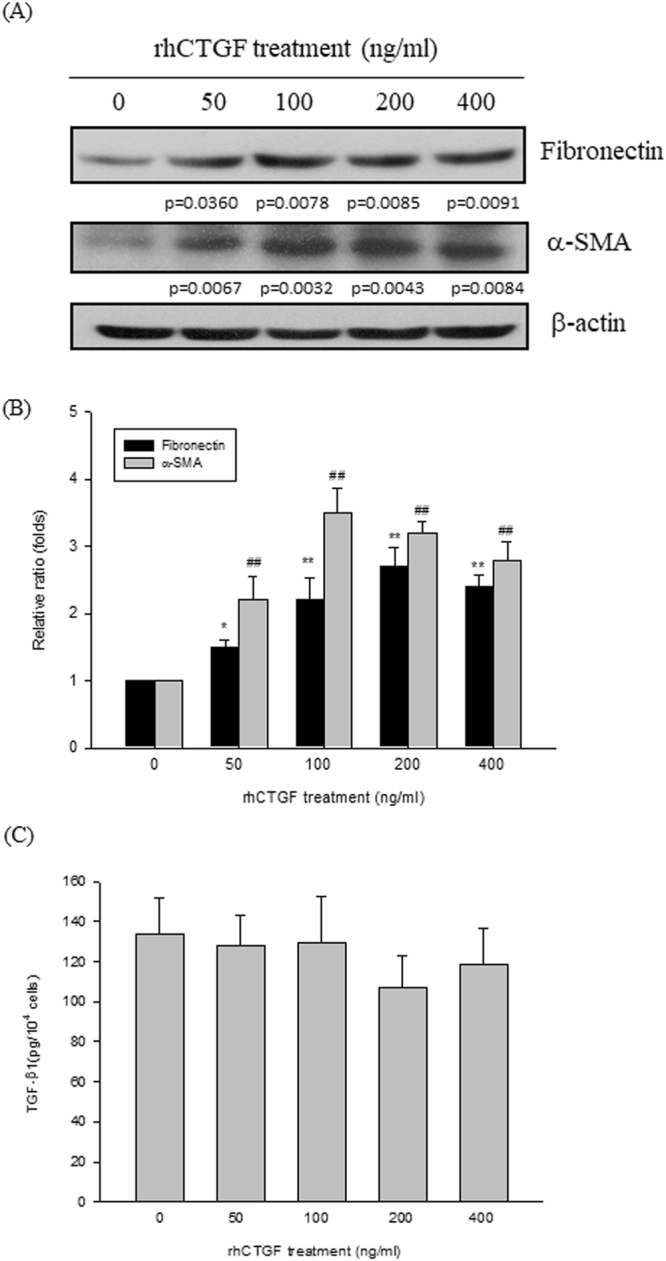
CTGF induced the fibronectin and α-SMA proteins expression in GO orbital fibroblasts. (**A**) After treatment of the orbital fibroblasts from GO1 patient with 50, 100, 200 and 400 ng/ml recombinant human protein CTGF (rhCTGF) for 24 hours, the proteins expression of fibronectin and α-SMA were examined by Western blots. (**B**) The representative bar graphic was constructed by the data obtained from three independent experiments, and data are presented as means ± S.D. (*p < 0.05; ** and ^##^p < 0.01). (**C**) By an ELISA kit, the intracellular levels of TGF-β1 in the orbital fibroblasts from GO1 patient was examined after rhCTGF treatment for 24 hours. The representative bar graphic was constructed by the data obtained from three independent experiments, and data are presented as means ± S.D. (*p < 0.05 vs. control without rhCTGF treatment; **^,##^p < 0.01 vs. control without rhCTGF treatment).

**Figure 5:**
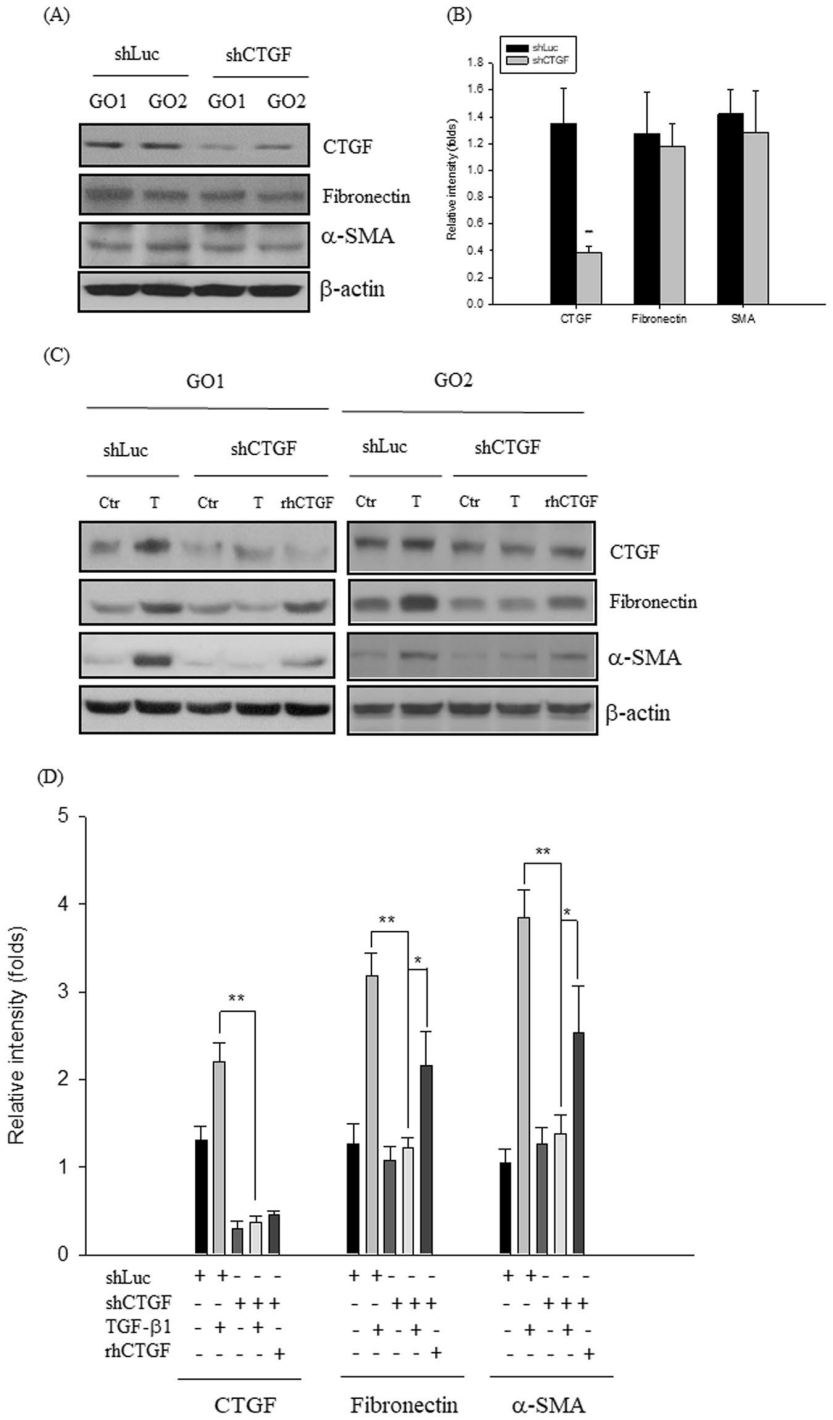
Knockdown of CTGF inhibited TGF-β1-induced the fibronectin and α-SMA proteins expression in the GO orbital fibroblasts. (**A**) By Western blots, the proteins expression of CTGF, fibronectin and α-SMA were observed in the orbital fibroblasts from GO patients (GO1 and GO2) with knockdown of luciferase (shLuc) and CTGF (shCTGF) genes, respectively. (**B**) The densitometric scan analysis of three independent Western blots for the folds change of proteins expression was constructed as a bar-graph. (**C**) After treatment of shLuc- and shCTGF-transfected orbital fibroblasts from GO patients (GO1 and GO2) with 5 ng/ml TGF-β1 or 100 ng/ml rhCTGF, respectively, the proteins expression of CTGF, fibronectin and α-SMA were analyzed by Western blots, and (**D**) the representative bar-graph was constructed from three independent experiments, and data are presented as means ± S.D. (*p < 0.05 vs. indicated groups; **p < 0.01 vs. indicated groups). The “shLuc” indicates the small hairpin RNA (shRNA) of luciferase, “shCTGF” indicates the shRNA of CTGF, “Ctr” indicates the control without TGF-β1 or rhCTGF treatment, and “T” indicates TGF-β1 treatment.

CTGF has been reported to play an important role in mediating the tissue remodeling and fibrosis in various cell types^19^. In order to clarify whether CTGF could contribute to the fibrotic response in GO orbital fibroblasts, we first treated the GO orbital fibroblasts with a recombinant human protein CTGF (rhCTGF). After treatment of the GO1 orbital fibroblasts with 50, 100, 200, and 400 ng/ml rhCTGF, respectively, for 24 hours, the protein expression levels of fibronectin and α-SMA were increased (p-value as indicated in Fig. 4A,B). We also noted that the intracellular levels of TGF-β1 was not significantly changed by the treatment of rhCTGF, suggesting that TGF-β1 signaling pathway was not involved in CTGF-induced increase of fibronectin and α-SMA proteins expression in the orbital fibroblasts from GO1 patient (Fig. 4C). To address specifically the role of CTGF, we transfected the GO orbital fibroblasts with a small hairpin RNA (shRNA) of CTGF gene (shCTGF) to knockdown the expression levels of CTGF protein in the GO orbital fibroblasts (GO1 and GO2). The shRNA of luciferase gene (shLuc) was used as a scramble control. Western blot analysis revealed that the expression of CTGF was decreased in cells transfected with shCTGF, but not in shLuc-transfected cells (p < 0.0001), and the inhibition of CTGF protein expression did not affect the expression of fibronectin and α-SMA proteins (p = 0.1883 and 0.1594, respectively, Fig. 5A,B). Most importantly, after treatment of shCTGF-transfected orbital fibroblasts with 5 ng/ml TGF-β1 for 24 hours, the TGF-β1-induced expression of CTGF, fibronectin and α-SMA proteins was inhibited (Fig. 5C,D). However, the protein expression levels of fibronectin and α-SMA could be induced significantly by the addition of 100 ng/ml rhCTGF in shCTGF-transfected GO orbital fibroblasts (Fig. 5C,D).

## Discussion

Orbital fibroblasts, one of the major target cells associated with GO, are involved in not only the early inflammatory process but also the subsequent tissue remodeling and fibrosis^20,21^. Fibroblast to myofibroblast transdifferentiation is mediated by a variety of inflammatory factors and mechanical stimuli^22,23^. TGF-β is the most important of these factors and has been implicated in numerous ocular fibrosis including corneal opacification, pterygium, posterior capsular opacification, proliferative vitreoretinopathy, fibrovascular membrane formation in proliferative diabetic retinopathy, subretinal fibrosis in neovascular age-related macular degeneration, and conjunctival fibrosis following glaucoma filtration surgery^24^. There are three isoforms of TGF-β, namely TGF-β1, TGF-β2 and TGFβ-3, have been identified in mammals. TGF-β1 has been shown to stimulate myofibroblast differentiation, extracellular matrix synthesis, or fibrosis in corneal-fibroblast^25^ conjunctival fibroblasts^26,27^, human retinal pigment epithelial cell^28^, and orbital fibroblasts^29^. TGF-β2 has also been reported to induce expression of extracellular matrix and/or fibrosis in cultured astrocytes of the human optic nerve head^30^, human Tenon’s fibroblasts^31^, and trabecular meshwork^32^. Current study revealed TGF-β1, but not TGF-β2, could induce CTGF, fibronectin, and α-SMA in Graves’ orbital fibroblasts. CTGF and fibronectin are essential mediators of extracellular matrix composition and α-SMA is a characteristic actin isoform expressed by myofibroblasts. Myofibroblasts transdifferentiation and increased extracellular matrix production in GO orbital fibroblasts represent important events of relevance to tissue remodeling and fibrosis in the pathogenesis of GO.

Moreover, we observed that the induction of protein expression of fibronectin and α-SMA by TGF-β1 in GO fibroblasts was significantly attenuated by CTGF knock-down with a shRNA transfection technique, which could be reversed by the addition of recombinant CTGF in shCTGF-transfected GO orbital fibroblasts. Studies in other cell types have also revealed the important roles of CTGF in the TGF-β-dependent induction of extracellular matrix production and myofibroblasts transdifferentiation^33–35^. It has been suggested that CTGF could act as an extracellular adapter protein by binding to TGF-β through its cysteine-rich domain and thus help to present them to their receptors to stimulate the fibrotic response^36^. In addition, CTGF has been reported to interact with various cytokines, growth factors, receptors, and matrix proteins and affect multiple signaling transduction pathways and processes important in tissue remodeling and fibrotic changes in organ structures^14,19,37^. Regardless of the complexity of the mechanisms by which CTGF modulates cell biology, several studies showed that inhibition of CTGF in liver, kidney, or the lungs has the potential to reverse tissue remodeling and the process of fibrosis^38–40^. TGF-β is a multipotent growth factor that exerts a wide range of normal physiological functions such as cellular proliferation, differentiation, apoptosis and extracellular matrix production. Therefore, clinical efforts to modulate the expression of TGF-β have been limited by concerns about its specificity of treatment effects and adverse reactions. CTGF acts as an important downstream mediator of TGF-β, thus it can provide a more efficient and safer target for suppression therapy as compared to TGF-β.

In this study, we also demonstrated the individual effect of CTGF on the induction of fibronectin and α-SMA proteins in GO orbital fibroblasts. Zhang *et al*. also demonstrated that CTGF could induce the transition of human tenon’s fibroblasts into myofibroblast individually without the initiation of exogenous TGF-β^41^. It means that CTGF not only exerts its effects as a mediator contributing to extracellular matrix production and myofibroblasts transdifferentiation after being induced by TGF-β, but also carries out its individual biological functions simultaneously.

Thyrotropin receptor (TSH-R) is the primary autoantigen in GO and recent studies have shown that TSH-R activation in orbital fibroblasts enhances hyaluronic acid synthesis and adipogenesis^42^. Thy-1 is a surface glycoprotein expressed on several different types of cells in the human body including orbital fibroblasts^43^. Increasing evidence revealed enhanced Thy-1 (CD90) expression in GO orbital tissues and fibroblasts^44^. TGF-β can trigger the myofibroblasts transdifferentiation of Thy-1(CD90)-positive orbital fibroblasts^12^. Recently, Fang *et al*. furtehr reported that interleukin (IL)-17A can promote TGF-β-induced fibrosis in CD90+ orbital fibroblasts^45,46^. However the biological effect of CTGF on this different subset of GO orbital fibroblasts and its relation to IL-17A needs further investigation.

Taken together, the results obtained from this study demonstrated that CTGF could promote myofibroblasts transdifferentiation and extracellular matrix production in GO orbital fibroblasts, either directly or by acting as a downstream mediator of TGF-β1. Therefore CTGF blockade could be a possible therapeutic target for amelioration of the tissue remodeling and fibrosis in the pathogenesis of GO.

## Electronic supplementary material

Supplementary Information
