# Supplementary material for: Essential role of connective tissue growth factor (CTGF) in transforming growth factor-β1 (TGF-β1)-induced myofibroblast transdifferentiation from Graves’ orbital fibroblasts
Source: Sci Rep. 2018 May 8;8:7276. doi: 10.1038/s41598-018-25370-3 (PMC5940888; doi:10.1038/s41598-018-25370-3)

Essential role of connective tissue growth factor (CTGF) in transforming growth factor-β1 (TGF-β1)-induced myofibroblast transdifferentiation from Graves' orbital fibroblasts

Chieh-Chih Tsai, MD, PhD, Shi-Bei Wu, PhD, Hui-Chuan Kau, MD, MS, Yau-Huei Wei. PhD

Original images of Western blots for Figure 1

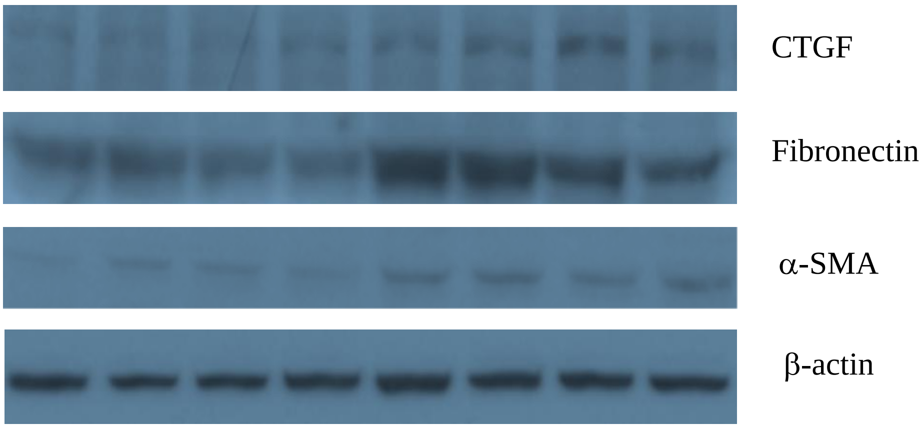

Original images of Western blots for Figure 2

(A)

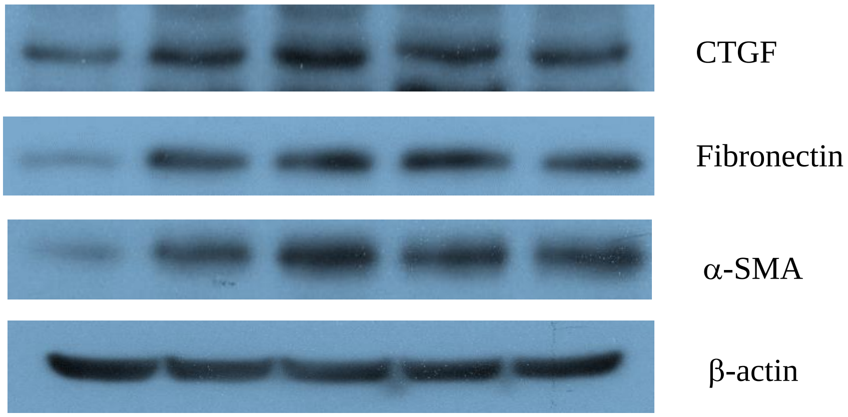

(B)

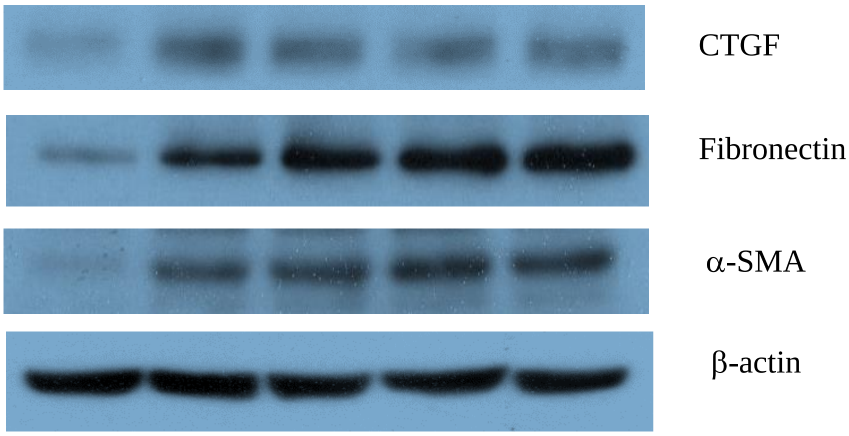

Original images of Western blots for Figure 2

(C)

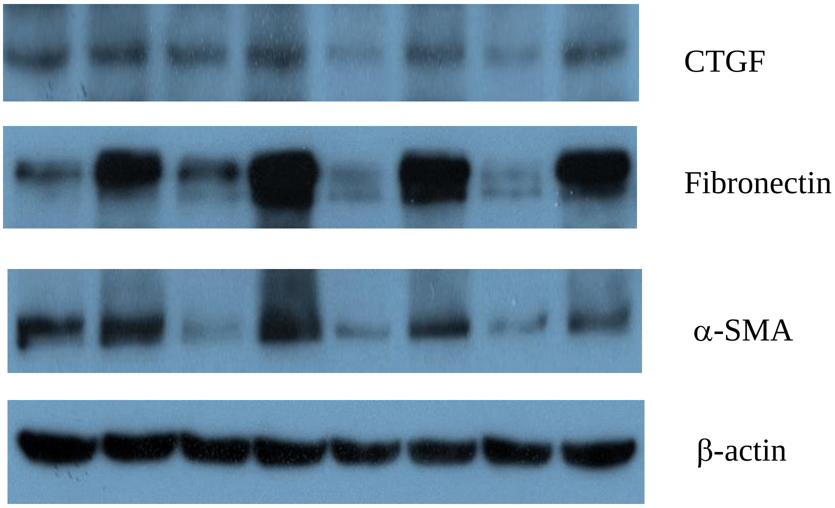

Original images of Western blots for Figure 3

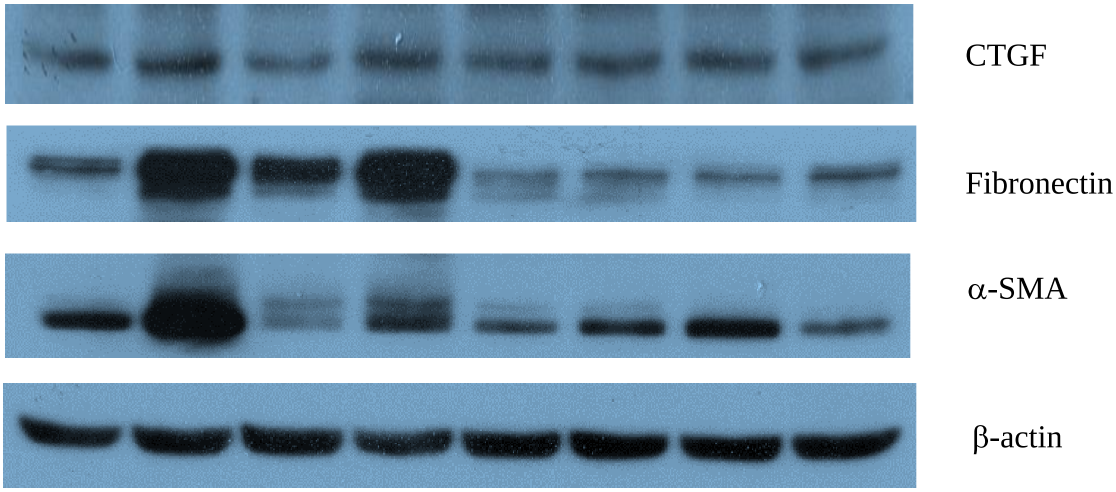

Original images of Western blots for Figure 4

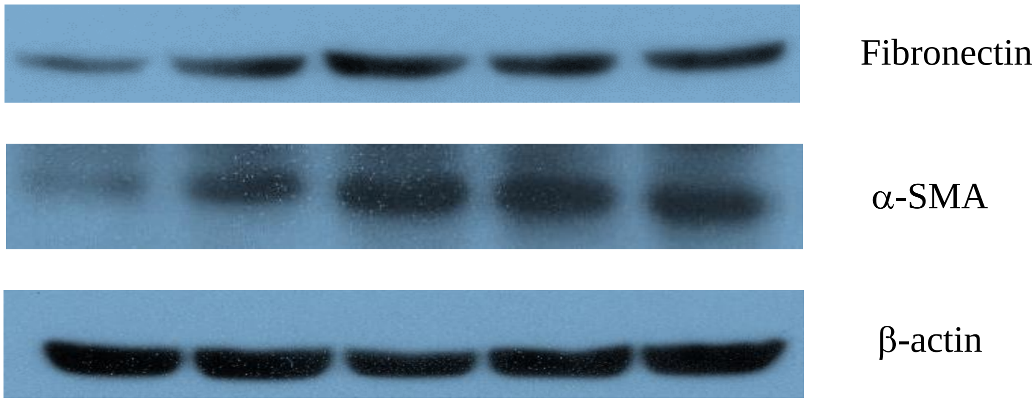

Original images of Western blots for Figure 5

(A)

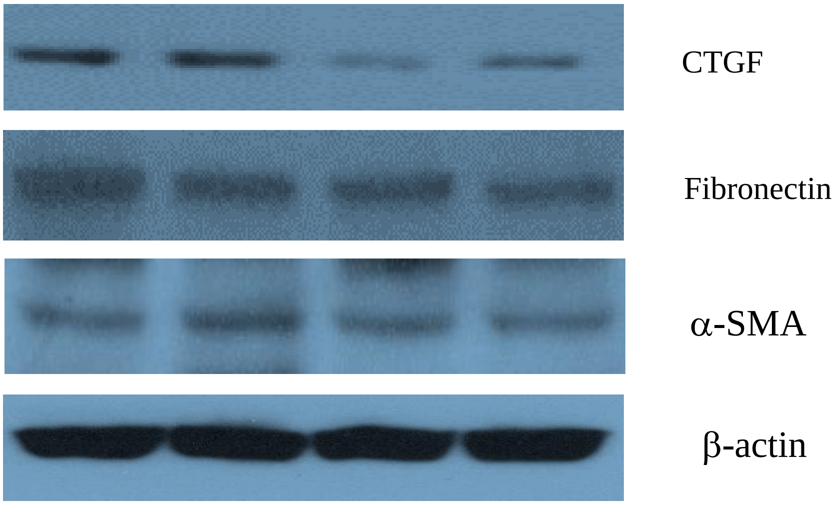

Original images of Western blots for Figure 5

(C) GO patient 1

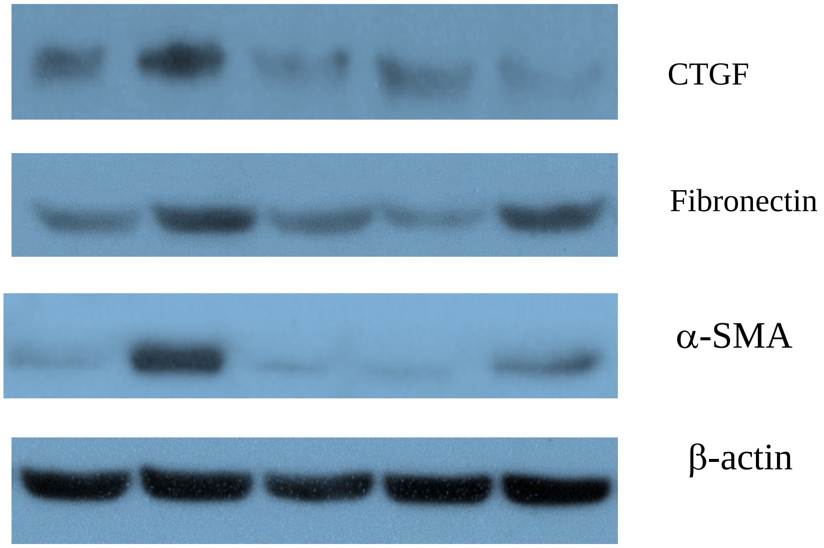

GO patient 2

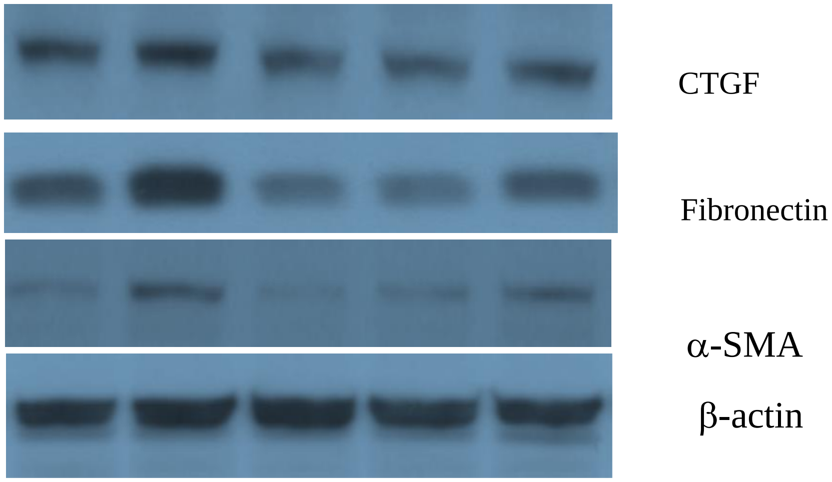

Supplement: Supplementary file 1 — Supplementary Information [file 41598_2018_25370_MOESM1_ESM.pdf]
